# Activation of the Hepcidin-Ferroportin1 pathway in the brain and astrocytic–neuronal crosstalk to counteract iron dyshomeostasis during aging

**DOI:** 10.1038/s41598-022-15812-4

**Published:** 2022-07-09

**Authors:** Mariarosa Mezzanotte, Giorgia Ammirata, Marina Boido, Serena Stanga, Antonella Roetto

**Affiliations:** 1 Department of Clinical and Biological Sciences, University of Turin, Turin, Italy; 2 Neuroscience Institute Cavalieri Ottolenghi, Department of Neuroscience Rita Levi Montalcini, University of Turin, Turin, Italy; 3 Present Address: Molecular Biotechnology Center Guido Tarone, University of Turin, Turin, Italy

**Keywords:** Biochemistry, Molecular biology, Neuroscience, Anatomy

## Abstract

During physiological aging, iron accumulates in the brain with a preferential distribution in regions that are more vulnerable to age-dependent neurodegeneration such as the cerebral cortex and hippocampus. In the brain of aged wild-type mice, alteration of the Brain Blood Barrier integrity, together with a marked inflammatory and oxidative state lead to increased permeability and deregulation of brain-iron homeostasis. In this context, we found that iron accumulation drives Hepcidin upregulation in the brain and the inhibition of the iron exporter Ferroportin1. We also observed the transcription and the increase of NCOA4 levels in the aged brain together with the increase of light-chain enriched ferritin heteropolymers, more efficient as iron chelators. Interestingly, in cerebral cortex and hippocampus, Ferroportin1 is mainly expressed by astrocytes, while the iron storage protein ferritin light-chain by neurons. This differential distribution suggests that astrocytes mediate iron shuttling in the nervous tissue and that neurons are unable to metabolize it. Our findings highlight for the first time that Hepcidin/Ferroportin1 axis and NCOA4 are directly involved in iron metabolism in mice brain during physiological aging as a response to a higher brain iron influx.

## Introduction

Iron is essential in many cellular and biological processes but it can also generate Reactive Oxidative Species (ROS) by Fenton reaction, contributing to the pathophysiology of many diseases^1^. Iron homeostasis is guaranteed by the action of proteins involved in iron import: Transferrin (Tf), Transferrin Receptors (TfR1), and Divalent Metal Transporter 1 (DMT1); iron export: Ferroportin 1 (Fpn1)^2^ and iron storage: cytosolic ferritin (Ft) heteropolymer, composed of 24 subunits of ferritin heavy (Ft-H) and light (Ft-L) chains^3^. However, the regulator of iron content and availability in the body is Hepcidin (Hepc), a peptide mainly produced by hepatocytes, that regulates iron levels by interacting with Fpn1. When body iron increases, Hepc rises as well and this causes Fpn1 degradation and, consequently, iron retention by the cells. So, Hepc lowers the amount of iron in the serum^1^, controlling intestinal iron uptake and release from splenic macrophages^4^, according to the body’s needs. The opposite situation occurs in iron deficiency conditions (i.e. anemia, hypoxia, ineffective erythropoiesis)^4,5^.

A new protein involved in iron metabolism is the Nuclear Receptor Coactivator 4 (NCOA4), a cargo protein able to promote selective autophagic ferritin degradation^6^. After NCOA4 binding to Ft-H, ferritin is carried to the lysosome and degraded and iron is released in the cytoplasm, modulating intracellular iron regulation, via “ferritinophagy”^7^. NCOA4 levels are in turn regulated by intracellular iron status^7^ and by the interaction with HERC2, an E3 ubiquitin-protein ligase^7,8^. In a NCOA4 knockout mouse model, it has been shown an iron phenotype with increased levels of Tf saturation, serum Ft and liver Hepc and an increase of Ft deposits in the liver and spleen^9^. Recently, an extra-hepatic function of NCOA4 was demonstrated^10^. However, up to now, no data are available on brain NCOA4 and Hepc/Fpn1 expression and function during aging or neurodegeneration.

In the brain, iron regulates important functions such as neurotransmission, myelination and division of neuronal cells^11^. Iron reaches the brain crossing the Blood Brain Barrier (BBB)^12^. Iron up-take is then mediated by TfR1 expressed on the luminal side of brain capillaries^13^. Once inside the cell, iron is released into the cytoplasmic space and exported through the abluminal membrane by unknown mechanisms in which Fpn1 and other transporters may be involved^14^.

It has been shown that Hepc is present in the brain, in mature astrocytes and oligodendrocytes^15^, where it plays a role in the control of iron amount together with its own iron regulatory proteins^14^. However, it is not yet clear whether the Hepc acting on Fpn1 in the brain is the one produced in the liver or not^15^. Although the peptide size and its amphipathic cationic structure^16^ would allow hepatic Hepc to pass the BBB, it has been shown that there is an endogenous cerebral Hepc expression^17^ and that it responds to brain iron state^18^.

Several conditions which are typical of aging such as inflammation, BBB damage due to the release of inflammatory mediators, free radicals and vascular endothelial growth factor^19^ cause iron redistribution and unbalance in the brain^20^.

With age, iron accumulates in the cerebral cortex (Ctx) and in the hippocampus (Hip), regions which are involved in neurodegenerative disorders^12^, but the underlying mechanism it is not yet known.

Here we demonstrated that NCOA4, Hepc and Fpn1 are activated in WT mice brain during physiological aging as a consequence of iron accumulation and that they participate to brain response to increased iron entry. Furthermore, we assessed the astrocytic–neuronal crosstalk and we found that the iron exporter Fpn1 co-localizes with astrocytes, while neurons are enriched in the iron deposit Ft-L heteropolymers, both in the Ctx and Hip. These data suggest that, while glial cells enhance iron export in the nervous tissue, neurons accumulate it, triggering neurodegenerative processes.

## Results

### Iron amount and distribution in the brain during aging correlates to the level of BBB permeability

**Figure 1:**
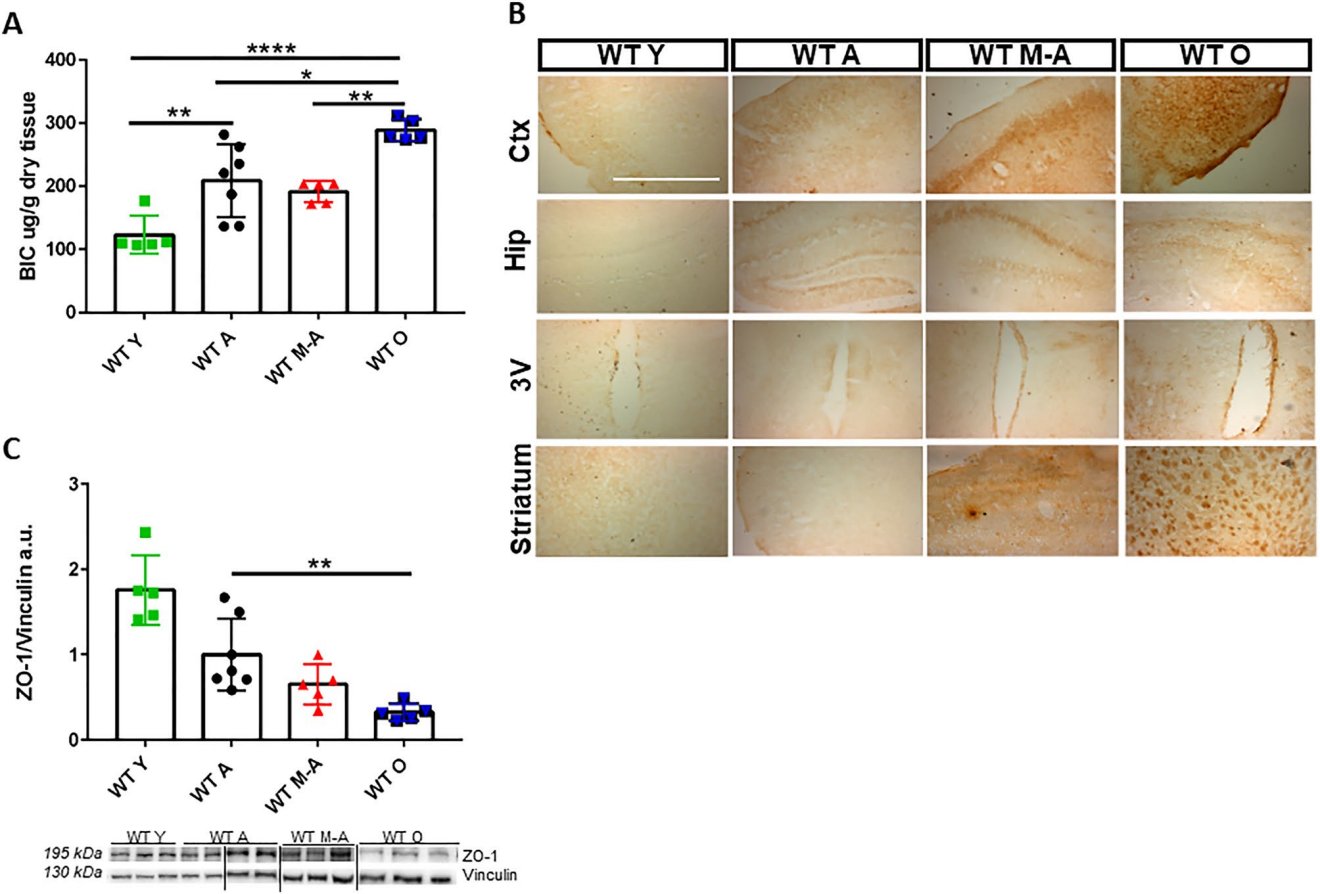
Iron accumulation in WT mice brain and impaired permeability of blood brain barrier (BBB). (**A**) Brain Iron Content (BIC) from mice total brain at different ages. (**B**) Sections of mice brain stained with DAB-enhanced Prussian Blue staining in cerebral cortex (Ctx), hippocampus (Hip), third ventricle (3v) and Striatum during aging. Scale bars:10X. Results on Liver Iron Content (LIC), Prussian Blue staining and serum iron are shown in Supplementary Figure 1S. (**C**) Western blotting analysis and quantification of ZO-1. Vertical black lines indicate image taken from different gels. Full length ZO-1 blot with a positive control (HeLa cells) is shown in Supplementary Figure 3S. *Statistically significant vs WT A control group *P < 0.05; **P < 0.01 ***P < 0.001 using two-tailed Student’s t-test. Number of analyzed mice: WT Y n = 5, WT A n = 7, WT M-A n = 5 and WT O n = 5.

Brain Iron Content (BIC) increases during aging at each experimental time point (Fig. 1A) in different brain areas as shown by histochemical analysis with DAB-enhanced Prussian blue Perls’ staining (Fig. 1B). WT O mice show an increased number of brown precipitates compared to WT A mice in specific parenchymal region such as Ctx, Hip CA regions, third ventricle (3 V) and striatum. Similarly, we also observed an age-dependent increase in the levels of iron in liver and in serum of old mice indicating a general perturbation of iron metabolism with physiological aging (Supplementary Figure 1S). Since progressive BBB damage is occurring not only in neurodegeneration^21^ but also during aging, we analysed Zonula occludens-1 (ZO-1) protein, whose role is to maintain the compactness of BBB acting as a bridge connecting Claudin and Occludin proteins to the actin cytoskeleton in order to stabilize the tight junction (TJ) structure^22^. ZO-1 levels significantly decrease during aging (Fig. 1C), therefore, we can hypothesize that age-dependent BBB altered permeability, could contribute, together with age-dependent metal dyshomeostasis, to iron accumulation in specific areas of the brain during physiological aging.

### Increased inflammatory and oxidative stress state during brain aging

**Figure 2:**
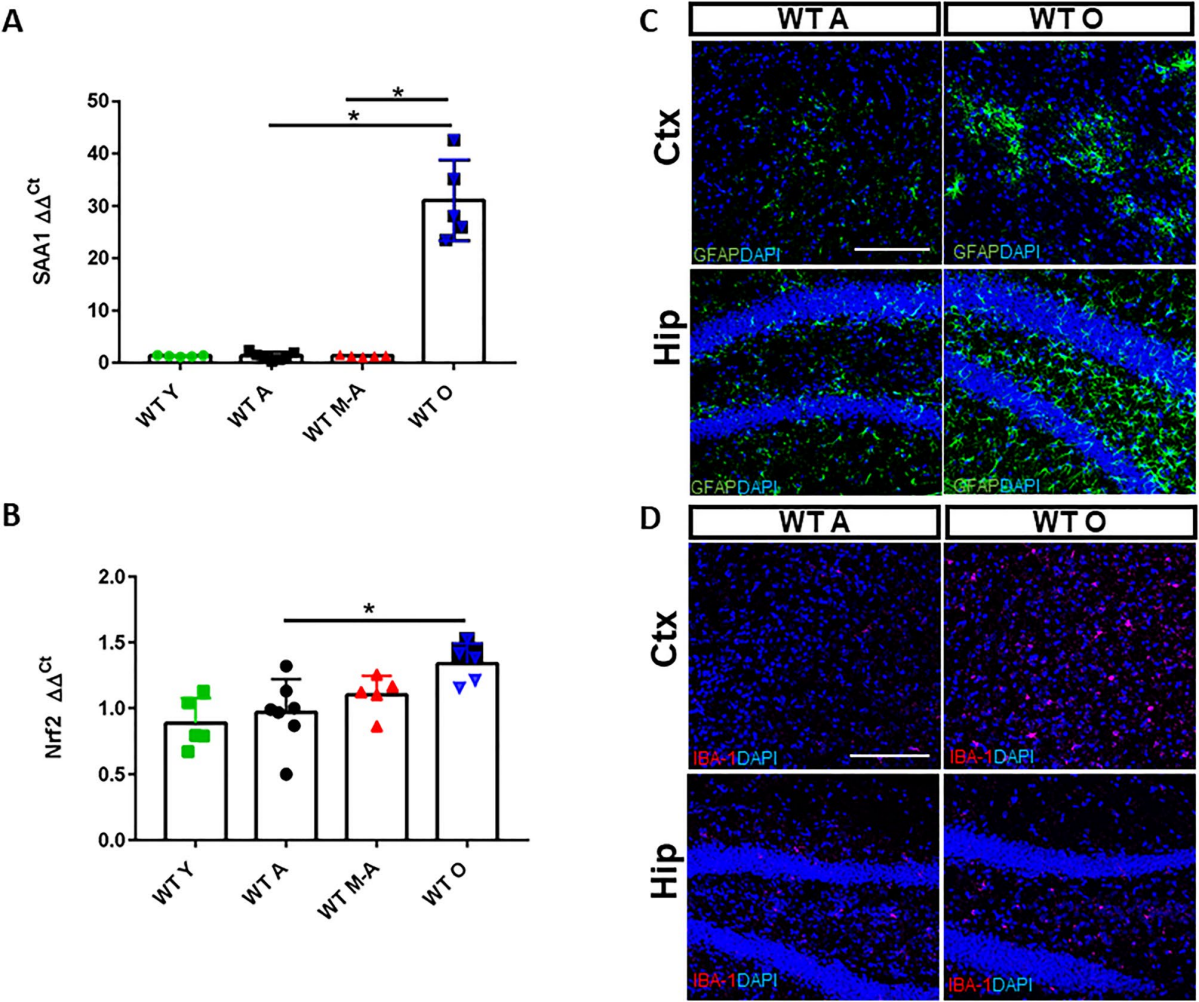
Iron-dependent inflammatory response and oxidative stress during aging. (**A**) Real-time PCR of SAA1 in total brain from all genotypes. (**B**) Nrf2 mRNA expression levels in total brain. The expression levels of the two genes were normalized to levels of β-glucuronidase (Gus-β) housekeeping gene (material and methods section). (**C**) Immunofluorescence anti-GFAP (green) and anti-IBA1 (pink) antibodies in cerebral cortex (Ctx) and hippocampus (Hip); 4,6-diamidino-2-phenylindole (DAPI) (blue) was used to counterstain cell nuclei. Scale bars:40X. *Statistically significant vs WT A control group *P < 0.05; **P < 0.01 ***P < 0.001 using OneWay ANOVA followed by Bonferroni’s post hoc analysis. Number of analyzed mice: (**A**–**B**): WT Y n = 5, WT A n = 7, WT M-A n = 5 and WT O n = 5; (**C**–**D**): WT A n = 5 and WT O n = 5.

The two main markers of neuroinflammation and oxidative stress, Serum amyloid A1 (SAA1) ^23^ and Nuclear factor erythroid 2-related factor 2 (Nrf2) ^24^, are overexpressed in aged brains. SAA1 expression levels is more than 20 times higher in WT O animals compared to WT A (Fig. 2A) and Nrf2 expression levels are constantly increasing during aging (Fig. 2B).

Moreover, we performed immunohistochemistry to selectively label reactive intermediate filament protein (GFAP)-positive astrocytes. In fact, GFAP is an indicator of neuroinflammation in the CNS^25^ and it is also involved in the progression of neurodegeneration in ischemia, AD, MS, Amyotrophic Lateral Sclerosis (ALS) and PD^26–28^. In addition, we also checked the expression of the Ionized calcium-binding adaptor molecule 1 (IBA-1), a microglia/macrophage-specific calcium-binding protein which is also a key molecule in proinflammatory processes^29^. We identified high astrocytes activation and an increased expression of microglia in both parenchymal regions of WT O mice where iron accumulated, Ctx and Hip, compared to those of WT A (Fig. 2C and D).

These data show that iron accumulation in the brain is accompanied by the neuroinflammatory and antioxidative stress response.

### Hepc/Fpn1 activation and ferritins response to iron accumulation during brain aging

**Figure 3:**
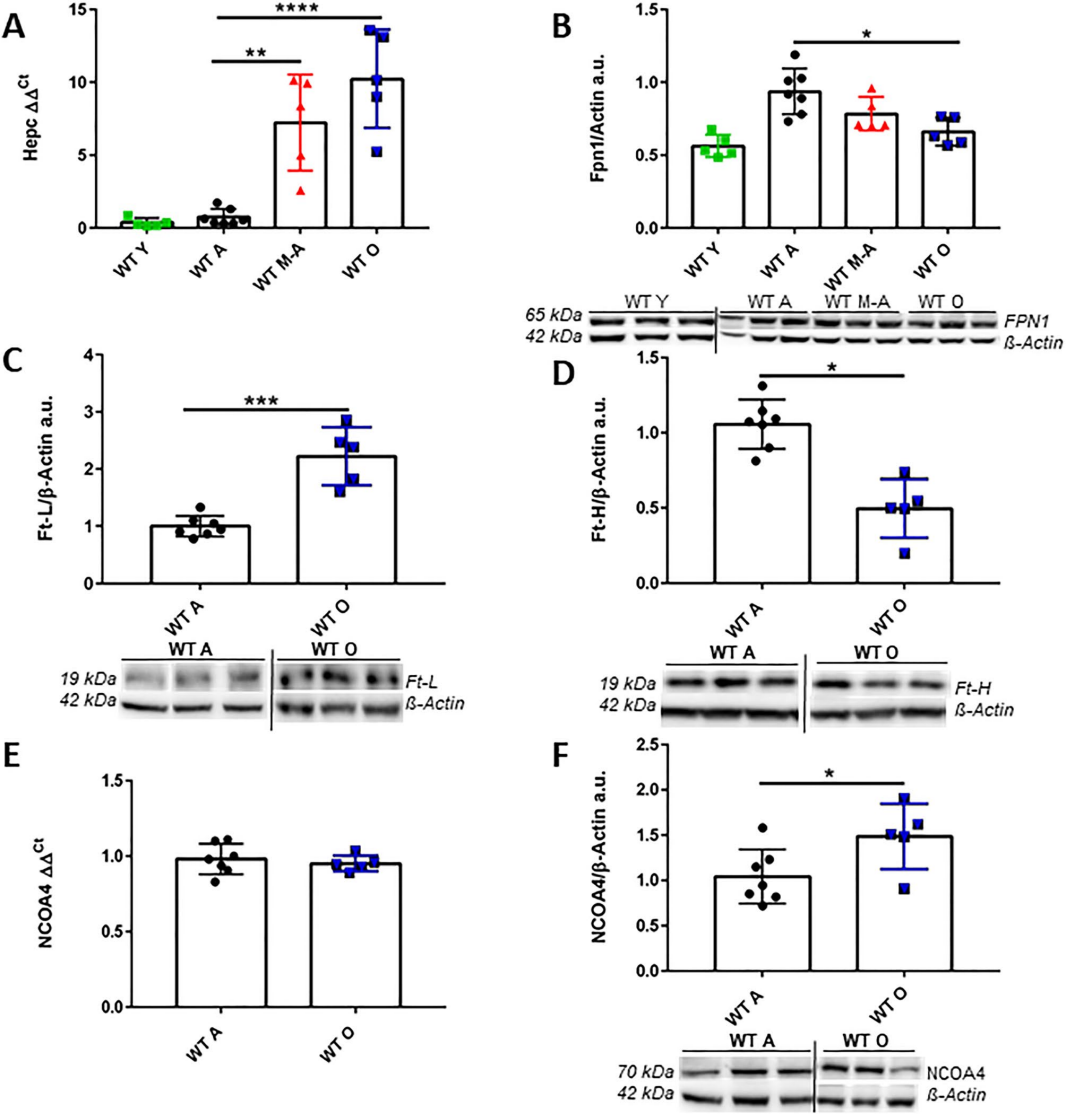
Hepcidin expression and iron transport/storage proteins quantification in mice brain. (**A**) Hepc transcription pattern. (**B**) Western blotting analysis and quantification of Fpn1. (**C**) Ft-L and (**D**) Ft-H. in mice total brain. (**E**) Real-time PCR of NCOA4 in total brain. The expression levels of NCOA4 were normalized to the levels of β-Glucuronidase (Gus-β) housekeeping gene (material and methods section) and (**F**) Western blotting analysis and quantification of NCOA4. Vertical black lines in blots images indicate that they are taken from different gels. Full length blots are presented in Supplementary Figure 4S. The full length blot with a positive control (liver) for Fpn1 is presented in Supplementary Figure 5S. *Statistically significant vs WT A control group *P < 0.05; **P < 0.01 ***P < 0.001 using OneWay ANOVA followed by Bonferroni’s post hoc analysis or two-tailed Student’s t-test. Number of analyzed mice: WT Y n = 5, WT A n = 7, WT M-A n = 5 and WT O n = 5.

In order to evaluate if the Hepc/Fpn1 axis has a role during brain physiologic aging, we measured both Hepc and Fpn1 in the whole brain of aged mice. Interestingly, we observed that Hepc gene expression significantly increases in WT M-A and WT O mice brain (Fig. 3A), while Fpn1 protein decreases (Fig. 3B). To investigate how neuronal cells responded to the increase of iron amount, we analysed in the total brain also the iron deposit protein ferritins (Ft) and separately evaluating the two polymers: ferritin light-chains (Ft-L) and ferritin heavy-chains (Ft-H). As expected, we observed a significant increase in Ft-L amount (Fig. 3C), but, surprisingly a 40% reduction of Ft-H in WT O animals' brains compared to the WT A (Fig. 3D). We also checked for NCOA4 levels of transcription and translation in the brain. NCOA4 gene results to be highly transcribed in the brain and its expression is comparable to that of the liver (Ct values 25 ± 1 and 24 ± 1.5 respectively) (Fig. 3E and^9^).

Furthermore, NCOA4 protein amount is also significantly increased in WT O mice brain compared to WT A (Fig. 3F).

Altogether, these results demonstrate that in old mice brain iron accumulation together with the inflammatory condition (Fig. 2A–C and D) induces Hepc expression and, consequently, Fpn1 degradation; therefore, activation of the Hepc/Fpn1 pathway in the brain promotes cellular iron retention. Furthermore, our results show for the first time that NCOA4 is transcribed in the brain and that it increases in WT O mice.

### Cellular distribution of iron deposits and export proteins in Ctx and Hip

**Figure 4:**
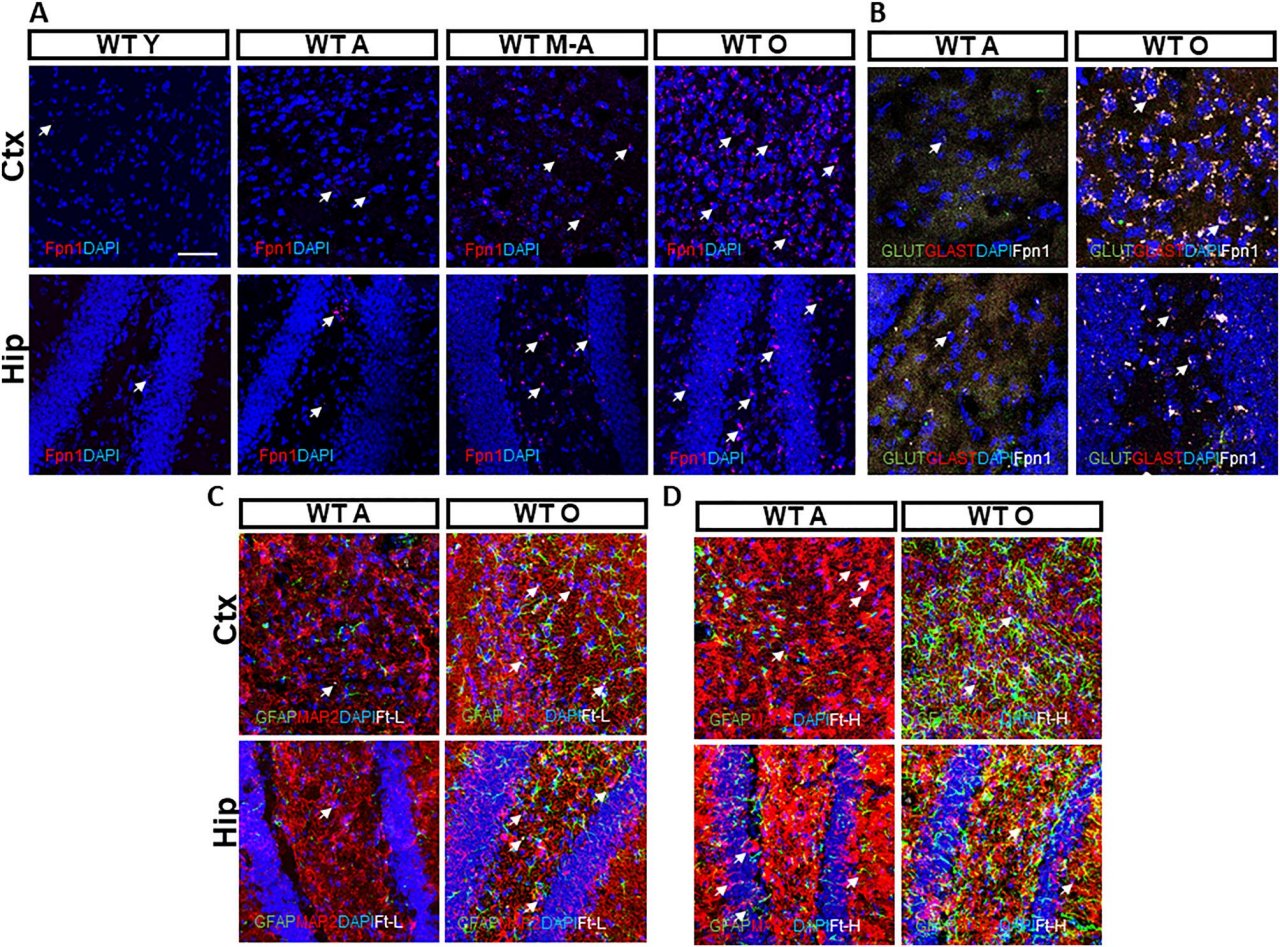
Ferroportin 1, Ferritin L and H chain protein cellular allocation in Ctx and Hip. (**A**) Immunofluorescence anti-Fpn1 antibody (red) in cerebral cortex (Ctx) and hippocampus (Hip). (**B**) Immunofluorescence of neuronal and astrocytic cells using anti-GLUT1 (green), anti-GLAST (red) and anti-Fpn1 antibodies in cerebral cortex (Ctx) and hippocampus (Hip). (**C, D**) Immunofluorescence of astrocytic and neuronal cells using anti-GFAP (green), anti-MAP2 (red), anti-Ft-L and anti-Ft-H antibodies in cerebral cortex (Ctx) and hippocampus (Hip). 4,6-diamidino-2-phenylindole (DAPI) (blue) was used to counterstain cell nuclei. Scale bars: 63X. Number of analyzed mice: WT Y n = 5, WT A n = 5, WT M-A n = 5 and WT O n = 5. Specifically, neuronal (MAP2) and Ferritins (Ft-L and Ft-H) localization are shown in Supplementary Figure 6S.

We decided to further analyse Fpn1 localization in mice brains. Immunofluorescence experiments revealed that Fpn1 is localized mainly in the Ctx and Hip at each age (Fig. 4A). Moreover, we checked for the cellular distribution of Fpn1 within the nervous tissue. When we co-labelled Fpn1 with specific astrocytic and neuronal markers, Glutamate Transporter (GLAST) and Vesicular Glutamate Transporter 1 (VGLUT1) respectively, we found that Fpn1 co-localizes with astrocytes (Fig. 4B).

Additionally, to discriminate if the accumulation of iron occurred specifically inside neurons and/or astrocytes, we co-stained Ft-L and Ft-H with Microtubule-Associated Protein 2 (MAP2) and GFAP, respectively. Compared to WT A, in WT O mice brains we observed a specific and marked increase of Ft-L deposits in cortical and hippocampal neurons but not in astrocytes (Fig. 4C). Ft-H isoform was also identified in the soma of cortical and hippocampal neurons (Fig. 4D), and resulted to be less abundant than Ft-L isoform in these cells. These results demonstrate that there is an “iron cross-talk” between astrocytes and neurons but they are participating differently in the process of iron distribution and metabolism/accumulation.

## Discussion

During aging and in neurodegenerative diseases with old age onset such as PD and AD, an increase in iron content was observed in multiple brain regions^30,31^. In pathological conditions, it was demonstrated to be the cause of motor deterioration^12^ and of proteins aggregation^32^ leading to cellular stress^33^. Parallel to deposition of iron in the brain, in the periphery, systemic iron levels decrease and old subjects are subjected to anemia^34^.

Systemic iron regulation is based on a complex protein regulatory system in which the hepatic Hepcidin (Hepc) plays a major role. Indeed, the iron dependent modulation of Hepc expression determines de facto iron availability in the body^35^. In the brain, iron homeostasis is regulated by the same proteins network that acts at the systemic level^30^ and the Hepc regulatory system is active also in the CNS^31^. Indeed, Hepc is expressed by glial cells and neurons from different brain regions and, under brain iron accumulation, it is activated and it induces Fpn1 decrease^36,37^. However, it is not clear yet whether this rely on brain or hepatic Hepc^15^. Moreover, it is not known how this regulatory system respond to intracerebral iron increase during aging. Intrigued by this question, we studied the brain expression of proteins involved in systemic iron homeostasis in wild type (WT) mice during aging.

We characterized the state of the brain at different ages by studying BBB integrity, brain inflammation and oxidative state, all key features related to the process of aging and that influence iron homeostasis (Figs. 1 and 2). It is known that BBB mitigates iron entry from the blood to the brain through highly regulated and selective systems: iron crosses the BBB bound to Tf through TfR-mediated endocytosis^38^ and brain vascular endothelial cells (BVECs) export intracellular iron using Fpn1, whose activity is conditioned by the iron ferroxidases ceruloplasmin and hephaestin^31^. Finally, iron is acquired by nervous cells through iron transporter proteins, as DMT1, and released from these cells through Fpn1^31^.

It is also known that iron accumulation in the brain, triggers the release of pro-inflammatory cytokines, determining an environment prone to neurodegeneration^39^.

Indeed, we demonstrated the progressive accumulation of iron during physiological aging in the Ctx, Hip, third ventricle and striatum and the parallel decrease of the BBB integrity. As a consequence of iron accumulation, the transcription of SAA-1, a protein related to acute inflammation and marker of neuroinflammation^40,41^, described also in AD as able to stimulate the release of cytokines and chemokines^23,41^, increases up to 1000 times in old mice brain. Moreover, the transcription of Nrf2, a redox-sensitive transcription factor^24^, is also increased, supporting the evidence of a stressful condition in WT O mice brain.

During aging, a consistent activation of astrocytes and a generalized neuroinflammation are evident^42^. In line with these findings, in both Ctx and Hip of WT O mice we observed high astrocytic and microglial activation.

Interestingly, in this context of increased iron deposition and inflammation in the brain, we found the activation of the Hepc/Fpn1 pathway: brain Hepc transcription increases and brain Fpn1 amount gradually decreases during aging. These observations are in line with what Sato and colleagues observed in the cerebral cortex and in mitochondria isolated from the brain of aged mice^43^. To better decipher the mechanism of the regulation of iron content in neuronal tissue during physiological aging, we also analyzed the iron deposit protein Ft and a newly characterized protein, NCOA4, since it is involved in Ft degradation and its inactivation in mice causes iron accumulation in the liver^9^. Specifically, NCOA4 promotes autophagic ferritin degradation through its binding to Ft-H subunit^7,44^. Ferritin levels are enhanced in a cellular model (HeLa cells) in which NCOA4 is silenced, suggesting that ferritin is constantly degraded by an NCOA4-dependent pathway^45^. Moreover, when NCOA4 knockdown is selectively targeting hepatocytes, the protein silencing promotes an increase in both iron amount and ferritin levels^46^.

Surprisingly, in old mice's brains we found an increased amount of NCOA4, contrary to what happens in the liver^9^. Furthermore, specifically evaluating the ferritin polymers, we observed an increase of Ft-L and a decrease of Ft-H chains in the aged mice brains. These data demonstrated that a differential Ft chains degradation occurs in both cortical and hippocampal neurons of old animals. We can suppose that Ft-L enriched heteropolymers are more efficient in iron chelation^3^ and are also more abundant in cortical and hippocampal neurons.

Interestingly, when we analysed Fpn1 localization in the brain by immunofluorescence, we found that Fpn1 colocalized with astrocytes, both in the Ctx and Hip. On the contrary, Ferritin accumulated in cortical and hippocampal neurons close to the soma, but not in astrocytes. We suggest that this could be due to a different detoxifying mechanism carried out by neurons and astrocytes, aimed either to store or remove iron excess, respectively. On the whole, this data revealed that aging dependent brain iron accumulation compromises the cells specific response: astrocytes, which are less susceptible than neurons to iron deposits-related toxicity^47^ and which play a protective role towards neurons^42^, have an increased iron export, while, neurons increase the metal storage in Ft-L rich heteropolymers. These deposits could trigger the neuronal death in Ctx and Hip evidenced during aging and even more during neurodegeneration^30^.

Furthermore, we showed for the first time that NCOA4 is transcribed in brain cells and that its expression is increased in WT O animals brain.

**Figure 5:**
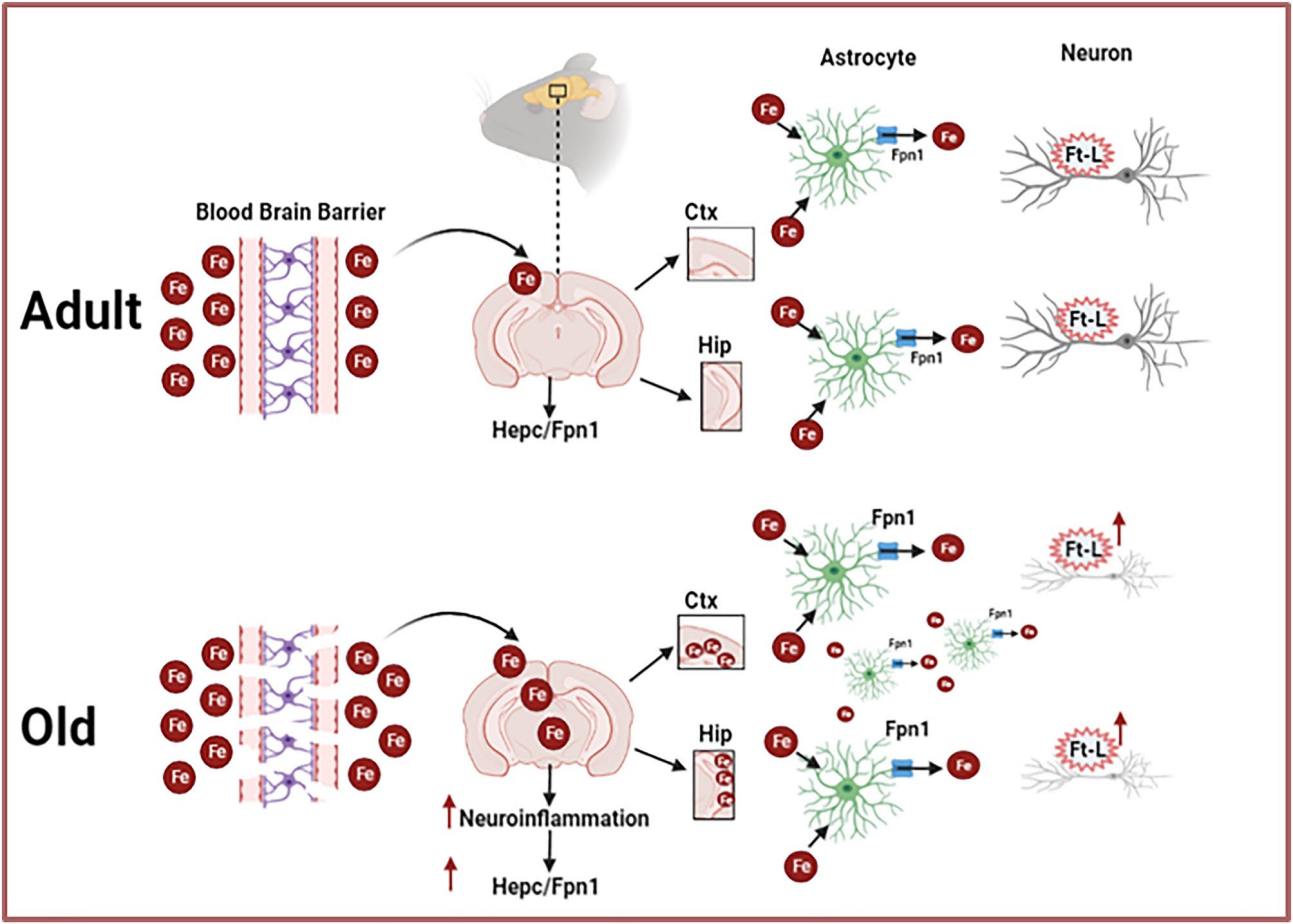
Iron regulation in the brain during aging. Schematic representation illustrating iron metabolism in old mice brains vs adults (see text for details). Fe: iron; Hepc: Hepcidin; Fpn1: Ferroportin 1; Ft-L: Ferritin-L; Ctx: cerebral cortex; Hip: hippocampus.

In conclusion, we demonstrated that even during physiologic aging, iron accumulates in the brain and that its accumulation, selectively localized in the Ctx and Hip, triggers neuroinflammation and the modification of the Hepc/Fpn1 pathway, all this enhancing iron availability imbalance and oxidative stress that could lead to neurodegeneration (Fig. 5).

In perspective, since NDs are characterized by inappropriate Hepc production^48^, a therapeutic approach aimed at modifying the Hepc response could be taken in consideration. Different strategies could be used, such as mini-Hepc and Hepc agonists^49,50^ by the stimulation/inhibition of Hepc production by targeting its regulators^35,48,51–53^. Additional research studies in animal models of NDs are required to clarify the CNS response to the increased iron aimed to exploit the results for the prevention and clinical management of patients with these diseases.

## Methods

### Animals

C57BL/6 J mice (WT) used for the study were purchased from the Jackson Laboratory and subdivided for age according to its classification (https://www.jax.org): until 2 months of age mice are considered Young (WT Y n = 5); from 2 to 6 months of age Adult (WT A n = 7); from 6 to 12 months of age Middle-aged (WT M-A n = 5) and from 12 months of age (between 18–24 months of age) Old (WT O n = 5) (Table 1S). Since from a pilot analysis on potential gender-related issues, no gender bias was observed (Supplementary Figure 2S), both male and female mice were analysed and grouped according to their age. Mice were housed in polycarbonate cages (Tecnoplast, Buggirate, Italy) provided with sawdust bedding, boxes/tunnels hideout as environmental enrichment. Food and water were provided ad libitum; environmental conditions were 12 h/12 h light/dark cycle, room temperature 24 °C ± 1 °C and room humidity 55% ± 5%. Each group of mice was fed with a Standard Diet (SD) (VRF1, Special Diets Services, Essex, United Kingdom). Mice were anaesthetized (ketamine, 100 mg/kg; Ketavet, Bayern, Leverkusen, Germany; xylazine, 5 mg/kg; Rompun; Bayer, Milan, Italy) and sacrificed by cervical dislocation. To performed histological analysis, a subset of at least n = 5 WT A and WT O mice were transcardially perfused with 4% paraformaldehyde (PFA) in phosphate buffered saline (PBS). Animals housing and all the experimental procedures were performed in accordance with European (Official Journal of the European Union L276 del 20/10/2010, Vol. 53, p. 33–80) and National Legislation (Gazzetta Ufficiale n° 61 del 14/03/2014, p. 2–68) for the protection of animals used for scientific purposes and the experimental procedure was approved by the Ethical Committee of the University of Turin and conducted according the ARRIVE guidelines.

### Real-time quantitative PCR

Total RNA from whole brain was extracted with TRIzol reagent. For reverse transcription, 2 μg of total RNA, 25 μM random hexamers and 100 U of reverse transcriptase (Applied Biosystems, California, USA) were used. Gene expression levels were measured using Real-time quantitative PCR in a CFX96 Real-time System (Bio-Rad, California, USA). For Nuclear factor erythroid 2-related factor 2 (Nfr2) and NCOA4 gene analysis, SYBR Green PCR technology (EVAGreen, Bio-Rad, California, USA) was used with specific primers (Supplementary Table 2S). For Hepc and Serum Amyloid A1 (SAA1) genes analysis, Taqman PCR method was used (Assays-on-Demand, Gene Expression Products, Applied Biosystems, California, USA). β-glucuronidase (Gus-β) was used as housekeeping control. Real-time quantitative PCR of the animals’ transcripts was carried out making duplicates of each n (n per group = min 3). The results were analyzed using the ΔΔCt method^54^.

### Immunoblotting

The Fpn1, Ft-H, Ft-L, NCOA4 and Zonula occludens-1 (ZO-1) proteins’ amount in the whole brain homogenates was evaluated by Western Blotting using specific antibodies. 50 μg of total brain lysates were separated on 6–12% SDS polyacrylamide gel and immunoblotted^55^. Primary antibodies Fpn1 (G-16), β-Actin (C-4), NCOA4 or ARA 70 (H-300) (Santa Cruz Biotechnology, Dallas, Texas, USA), ZO-1 (GeneTex, California, USA) and Vinculin (Invitrogen, Massachusetts, USA) were used. Antibodies used to detect Ft-H and Ft-L were provided by Sonia Levi, University of Vita Salute, Milan, Italy. Data were normalized on β-Actin or Vinculin amount in the same samples (Image Lab 4.0.1 Software, Bio-Rad, California, USA)^56^. Complete reference to all the antibodies is reported in Supplementary List 1.

### Immunofluorescence

Animals were perfused, brains were removed, post-fixed in PFA for 24 h at 4 °C and cryoprotected in 30% sucrose in 0.12 M phosphate buffer^57^. Brains were cut in 30 μm thick coronal sections collected in PBS and then stained to detect: Fpn1 (G-16, Santa Cruz Biotechnology, Dallas, Texas, USA), Ft-L, Ft-H (S. Levi, University of Vita Salute, Milan), Glial Fibrillary Acidic Protein (GFAP) (Dako, California, United States), Microtubule-Associated Protein 2 (MAP2) (Merck Millipore Burlington, Massachusetts, United States), Vesicular Glutamate Transporter 1 (VGLUT1) (Merck Millipore Burlington, Massachusetts, United States), Glutamate Transporter (GLAST) (Thermo Fischer Scientific Waltham, Massachusetts, United States) and Ionized calcium-binding adaptor molecule 1 (IBA-1) (Abcam, Cambridge, United Kingdom). After overnight incubation at 4 °C in PBS with 2% normal donkey serum (NDS)^58^, sections were exposed to Cy2-, Cy3- (Jackson ImmunoResearch Laboratories, West Grove, PA) and 647 Alexa Fluor-conjugated secondary antibodies (Molecular Probes Inc, Eugene Oregon) for 1 h at room temperature. DAPI (4,6-diamidino-2-phenylindole, Fluka, Italy) was used to counterstain cell nuclei. After processing, sections were mounted with Tris-glycerol supplemented with 10% Mowiol (Calbiochem, LaJolla, CA). The samples were examined by a Leica TCS SP5 confocal laser scanning microscope (Leica, Mannheim); z-stacks images were taken at 40X and 63X magnification.

### Iron parameters

Brain nonheme iron content (BIC) was evaluated using 20 mg of dissected and dried murine whole brains^59^. Perfused brains were stained for nonheme ferrous iron by Prussian blue Perl’s using a commercial kit (Bio-Optica, Milan, Italy). To improve the sensitivity, an intensification step with DAB (3-3′-diaminobenzidine tetrahydrochloride)^60^ was performed. Images were taken at 10X magnification using a Leica DM4000B automated microscope with IM50 program for acquisition (Leica Microsystems, Wetzlar, Germany).

### Statistical analysis

One-way ANOVA followed by Bonferroni’s post hoc analysis or two-tailed Student’s t-test were applied according to the experimental group’s number. P values of < 0.05 were considered as statistically significant. Analyses were performed with Image Lab 4.0.1 and GraphPad Prism 7.00. Data were expressed as average ± SD of the mean. Significance was defined as *P < 0.05, **P < 0.01 and ***P < 0.001. WT adult (A) mice were used as normalizer. The number of samples in each experimental condition is indicated in the figure legends. In each Western Blotting experiment, we reported 3 samples per group.

## Supplementary Information

Supplementary Information.

## Data Availability

The data regarding reference genes for Nrf2, NCOA4 and Gus-B primers are openly available in the repository “Nucleotide” at https://www.ncbi.nlm.nih.gov/nuccore, reference numbers NM_010902.4, NM_019744.4 and NM_010368.2.
