# Supplementary material for: Activation of the Hepcidin-Ferroportin1 pathway in the brain and astrocytic–neuronal crosstalk to counteract iron dyshomeostasis during aging
Source: Sci Rep. 2022 Jul 9;12:11724. doi: 10.1038/s41598-022-15812-4 (PMC9271044; doi:10.1038/s41598-022-15812-4)
Supplement: Supplementary file 1 — Supplementary Information. [file 41598_2022_15812_MOESM1_ESM.pdf]

Activation of the Hepcidin-Ferroportin1 pathway in the brain and astrocytic-neuronal crosstalk to counteract iron dyshomeostasis during aging

Mariarosa Mezzanotte[1], Giorgia Ammirata[1,3], Marina Boido[2], Serena Stanga[2,†,*] and Antonella Roetto[1,†,*]

[1]Department of Clinical and Biological Sciences, University of Turin, Italy;
[2]Neuroscience Institute Cavalieri Ottolenghi, Department of Neuroscience Rita Levi Montalcini, University of Turin, Italy
[3]Current affiliation: Molecular Biotechnology Center Guido Tarone, University of Turin, Italy
[†]These authors contributed equally to this study

*Correspondence: Serena Stanga serena.stanga@unito.it and Antonella Roetto antonella.roetto@unito.it

Supplementary Table 1.

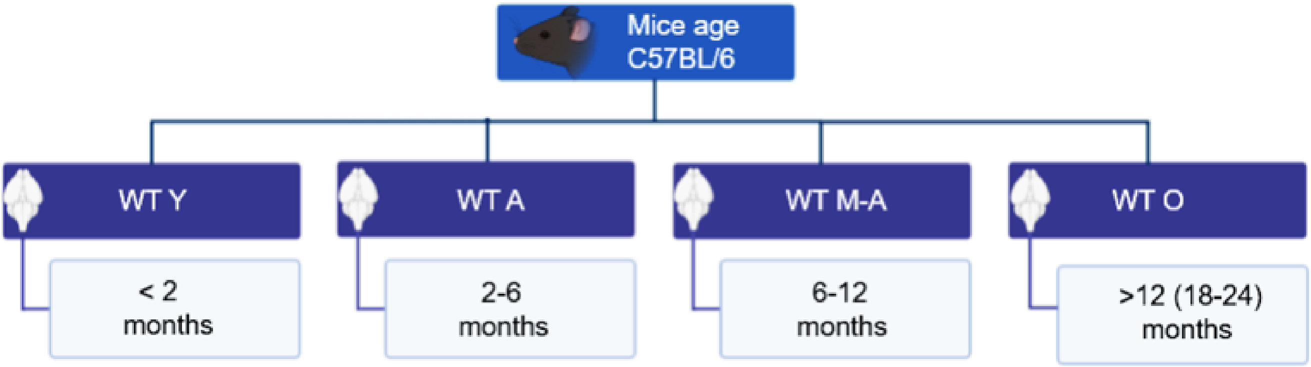

Table 1S: Age and number of mice analyzed in the study

The Jackson Laboratory classification (https://www.jax.org): WT Y, Young (n=5); WT A, Adult (n=7); WT M-A, Middle-Aged (n=5); WT O, Old (n=5).

Supplementary Figure 1.

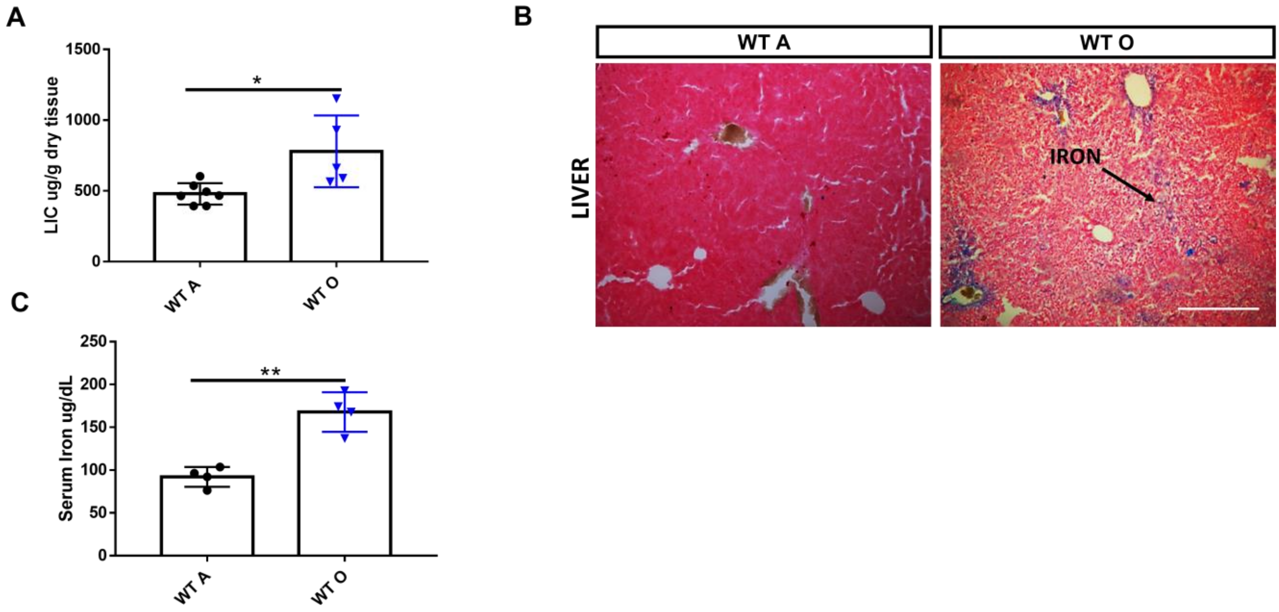

Figure 1S: Liver and serum iron content.

(**A**) Liver Iron Content (LIC) from Adult and Old mice. (**B**) Sections of mice livers stained with Prussian Blue. Scale bars:10X. (**C**) Serum iron amount from WT A (Adult) and WT O (Old) mice. For serum iron analysis, mice were anaesthetised (ketamine, 100 mg/kg; Ketavet, Bayern, Leverkusen, Germany; xylazine, 5mg/kf; Rompun; Bayer, Milan, Italy) and blood were collected from retro-orbital sinus of mice using a sterile hematocrit tube. Collected blood samples were centrifuged at 3000 g for 10 minutes and serum was taken. Serum iron levels were evaluated by using a commercial kit, Iron Direct Method (Biolabo, Mainz, France), following the manufacturer's instructions. The absorbance was measured at 600 nm via a spectrophotometer. *Statistically significant vs WT A control group *P <0.05; **P <0.01 ***P <0.001 using two-tailed Student's t-test.

Supplementary Table 2.

Genes	Forward Primers (5'-3')	Reverse Primers (5'-3')
**Nrf2**	ACTTGGAGTTGCCACCG	TTCTCCTGTTCCTTCTGGAG
**NCOA4**	GCTCCTCAAGTATTGGG	GAAGCCACTCACTCAGAG
**Gus-β**	GGGACCATCGTCTACAAGACTGA	GCTTGTGTCCTGGACAAAGTAACC

Table 2S: Primers sequences.

The sequences of primers that have been used for amplification are reported in the table. Genes are abbreviated as follows: Nuclear factor erythroid 2-related factor 2 (Nrf2), Nuclear receptor coactivator 4 (NCOA4); β-glucuronidase (Gusb-β). Primers for Nrf2, NCOA4, and Gus-β amplification were designed on the following mRNA sequence: NM_010902.4, NM_019744.4, and NM_010368.2. Primers efficiency was calculated with qRT–PCR (CFX96, BioRad, Hercules, CA, USA) by using serial dilutions of a template cDNAs. Primers showed an efficiency between 90 and 110%. The specificity of each amplicon was evaluated by analyzing the melting curve.

Supplementary List 1 of antibodies used by Western blotting.

1. **Fpn1** polyclonal anti-goat, dilution used: 1:1000.

- Deng Q, Yang S, Sun L, et al. Salmonella effector SpvB aggravates dysregulation of systemic iron metabolism via modulating the hepcidin-ferroportin axis. Gut Microbes. 2021;13(1):1-18. doi:10.1080/19490976.2020.1849996
- Yoshida M, Minagawa S et al. Involvement of cigarette smoke-induced epithelial cell ferroptosis in COPD pathogenesis. Nat Commun. 2019 Jul 17;10(1):3145. doi: 10.1038/s41467-019-10991-7
- Pellegrino RM, Boda E, Montarolo F, Boero M, Mezzanotte M, Saglio G, Buffo A, Roetto A. Transferrin Receptor 2 Dependent Alterations of Brain Iron Metabolism Affect Anxiety Circuits in the Mouse. Sci Rep. 2016 Aug 1;6:30725. doi: 10.1038/srep30725. PMID: 27477597; PMCID: PMC4967901.

2. **ZO1** polyclonal anti-rabbit, dilution used: 1:1000.

- De Tomi E, Campagnari R, Orlandi E, Cardile A, Zanrè V, Menegazzi M, Gomez-Lira M, Gotte G. Upregulation of miR-34a-5p, miR-20a-3p and miR-29a-3p by Onconase in A375 Melanoma Cells Correlates with the Downregulation of Specific Onco-Proteins. Int J Mol Sci2022 Jan 31;23(3):1647. doi: 10.3390/ijms23031647
- HyunA Jo, Dahyun Hwang, Jeong-Keun Kim, Young-Hee Lim. Oxyresveratrol improves tight junction integrity through the PKC and MAPK signaling pathways in Caco-2 cells. Food Chem Toxicol 2017 Oct;108(Pt A):203-213. doi: 10.1016/j.fct.2017.08.002.

3. **NCOA4** polyclonal anti-rabbit, dilution used: 1:1000.

- Kollara A, Ringuette MJ, Brown TJ. Dynamic distribution of nuclear coactivator 4 during mitosis: association with mitotic apparatus and midbodies. PLoS One. 2011;6(7):e22257. doi: 10.1371/journal.pone.0022257.

4. **Ft-L** and **Ft-H** polyclonal antibodies anti-rabbit, dilutions used: 1:1000.

- Pellegrino RM, Boda E, Montarolo F, Boero M, Mezzanotte M, Saglio G, Buffo A, Roetto A. Transferrin Receptor 2 Dependent Alterations of Brain Iron Metabolism Affect Anxiety Circuits in the Mouse. Sci Rep 2016 Aug 1;6:30725. doi: 10.1038/srep30725;

- Santambrogio P, Cozzi A, Levi S, Rovida E, Magni F, Albertini A, Arosio P. Functional and immunological analysis of recombinant mouse H- and L-ferritins from Escherichia coli. Protein Expr Purif 2000 Jun;19(1):212-8. doi: 10.1006/prep.2000.1212.

Supplementary Figure 2.

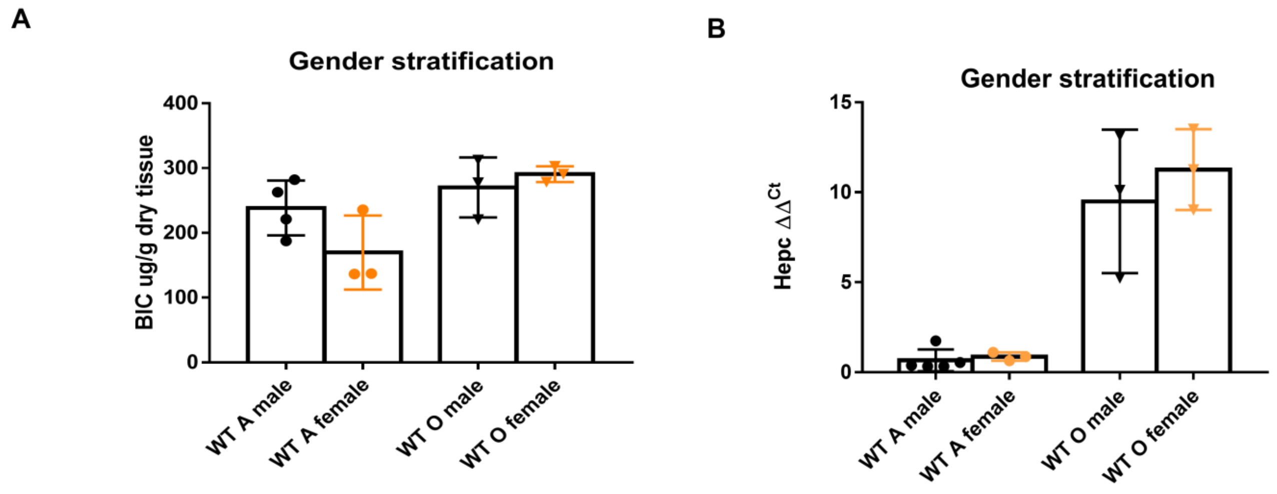

Figure 2S: Gender stratification.

(**A**) Brain Iron Content (BIC) parameters and (**B**) Hepc expression: gender stratification between WT A (Adult) *vs* WT O (Old) males and females.

Supplementary Figure 3.

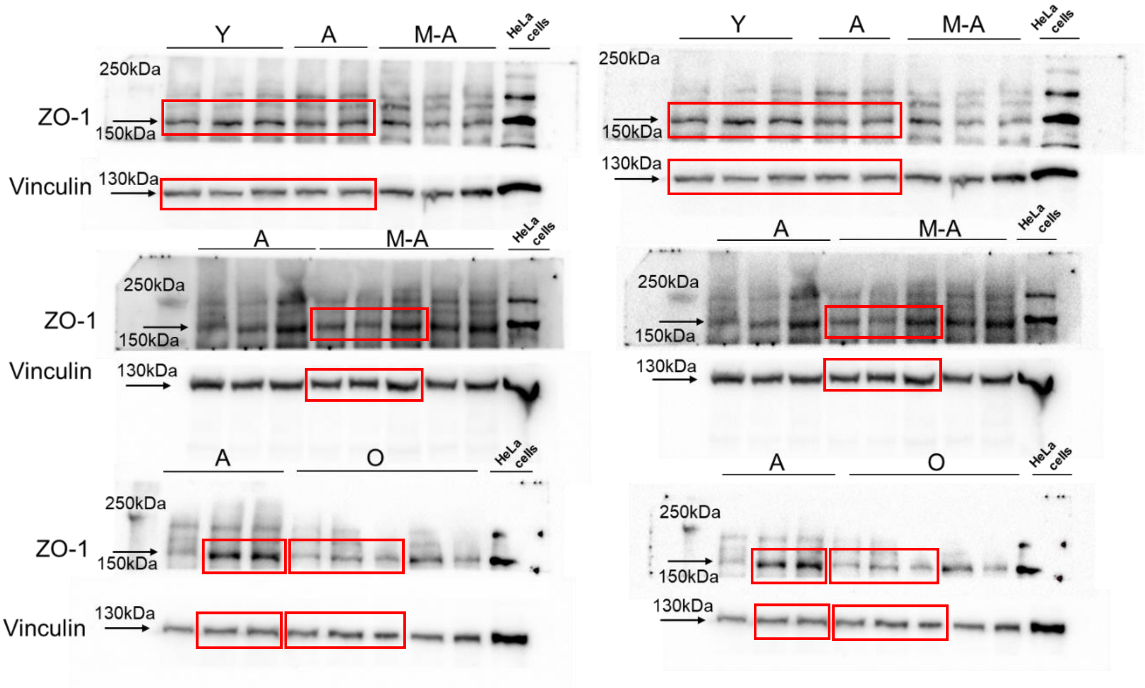

Figure 3S: Zonula occludents-1 protein quantification in the total brain from all age groups.

Full-length western blottings of ZO-1 protein (ZO-1). HeLa cells were used as a positive control. Blots were cut prior to over night hybridization with primary antibodies according to their molecular weight: 195 kDa for ZO-1 and 130 kDa for Vinculin. The regions of the original blots used in main figures are denoted by red boxes. On the right, we reported images with low contrast. Data were normalized on Vinculin amount in the same samples (Image Lab 4.0.1 Software, Bio-Rad, California, USA).

Supplementary Figure 4.

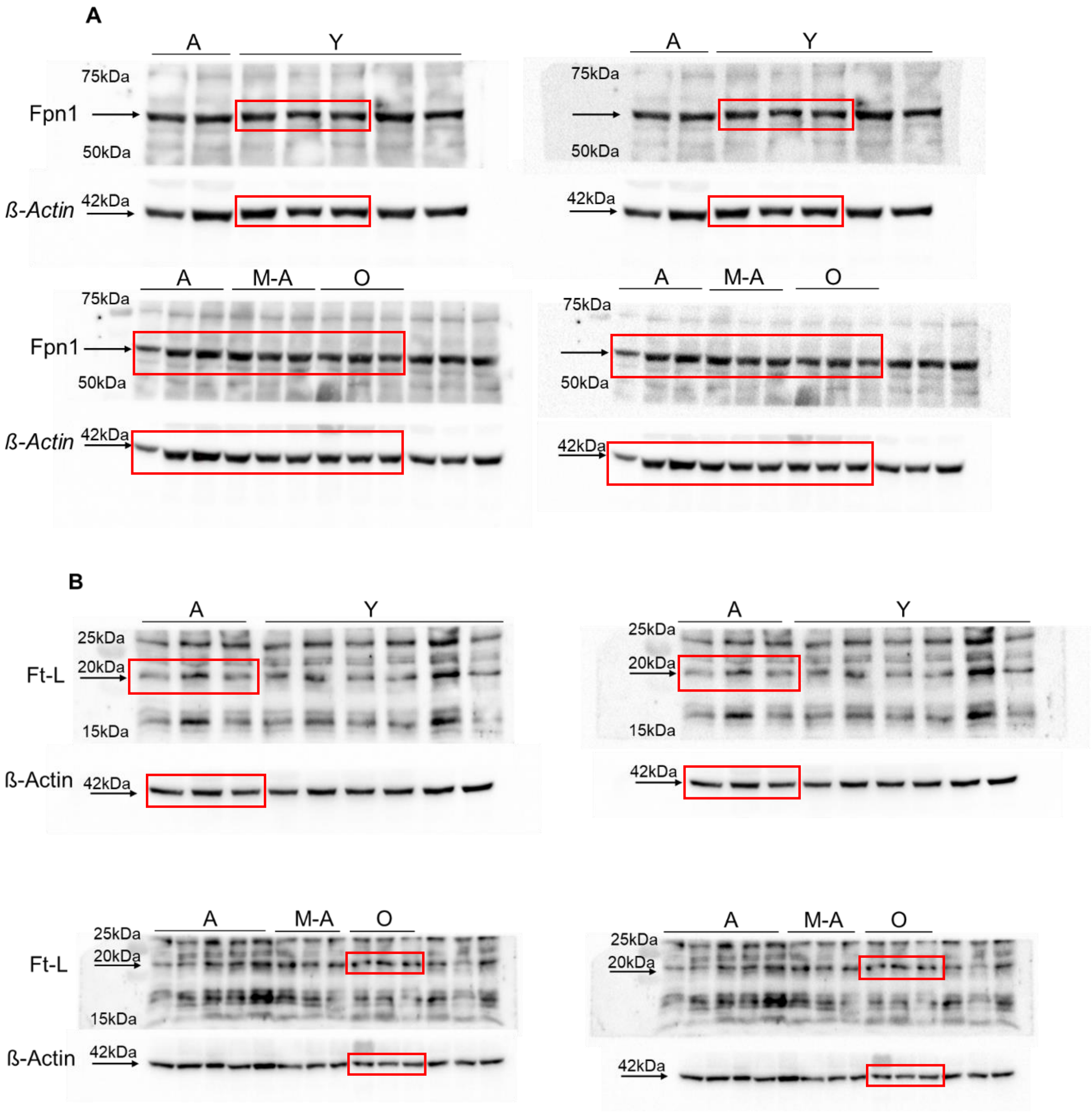

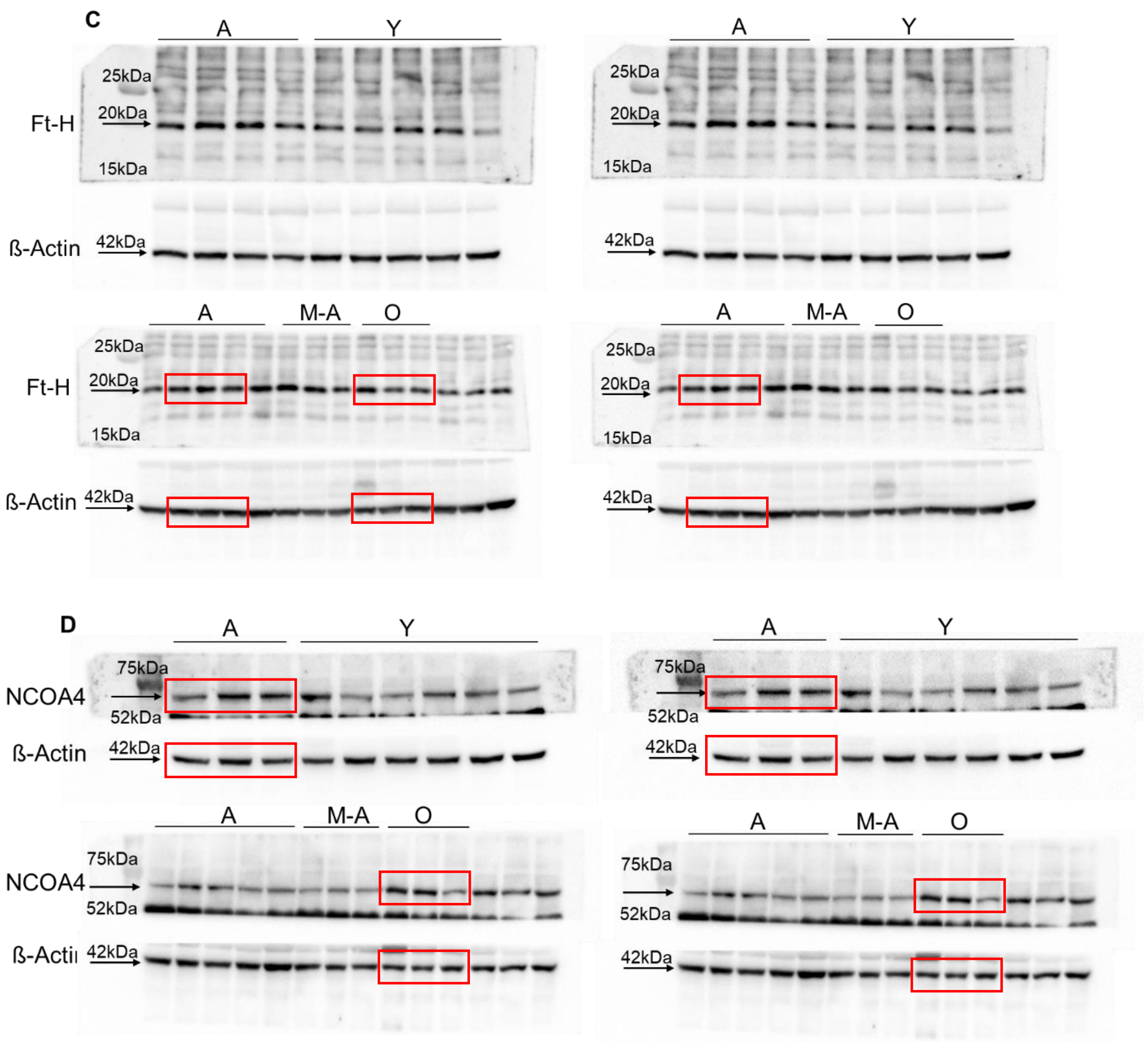

Figure 4S: Iron proteins quantification in the total brain from all age groups.

Full-length western blottings of (**A**) Ferroportin 1 (Fpn1), (**B**) Ferritin-L (Ft-L), (**C**) Ferritin-H (Ft-H) and (**D**) Nuclear receptor coactivator 4 (NCOA4). Data were normalized on the β-Actin amount in the same samples (Image Lab 4.0.1 Software, Bio-Rad, California, USA. Blots were cut prior to over night hybridization with primary antibodies according to their molecular weight: 65 kDa for Fpn1, 19 kDa for Ft-L and Ft-H, 70 kDa for NCOA4 and 42 kDa for β-actin. The regions of the original blots used in the main figures are denoted by red boxes. On the right, we reported images with low contrast.

Supplementary Figure 5.

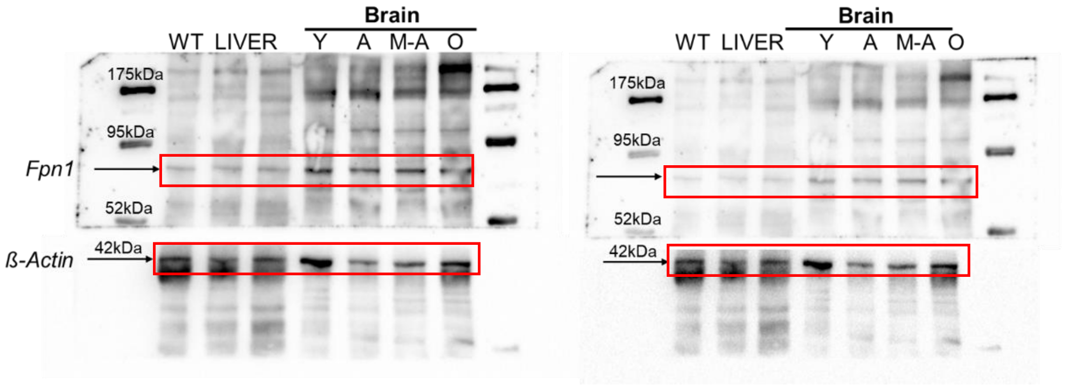

Figure 5S: Ferroportin 1 protein in total liver and brain from all age groups.

Full-length western blottings of Ferroportin 1 (Fpn1). Wild-type mouse livers were used as a positive control. Blots were cut prior to oven nigh hybridization with primary antibodies according to their molecular weight: 65 kDa for Fpn1 and 42 kDa for β-actin. The regions of the original blots used in main figures are denoted by red boxes. On the right, we reported images with low contrast.

Supplementary Figure 6.

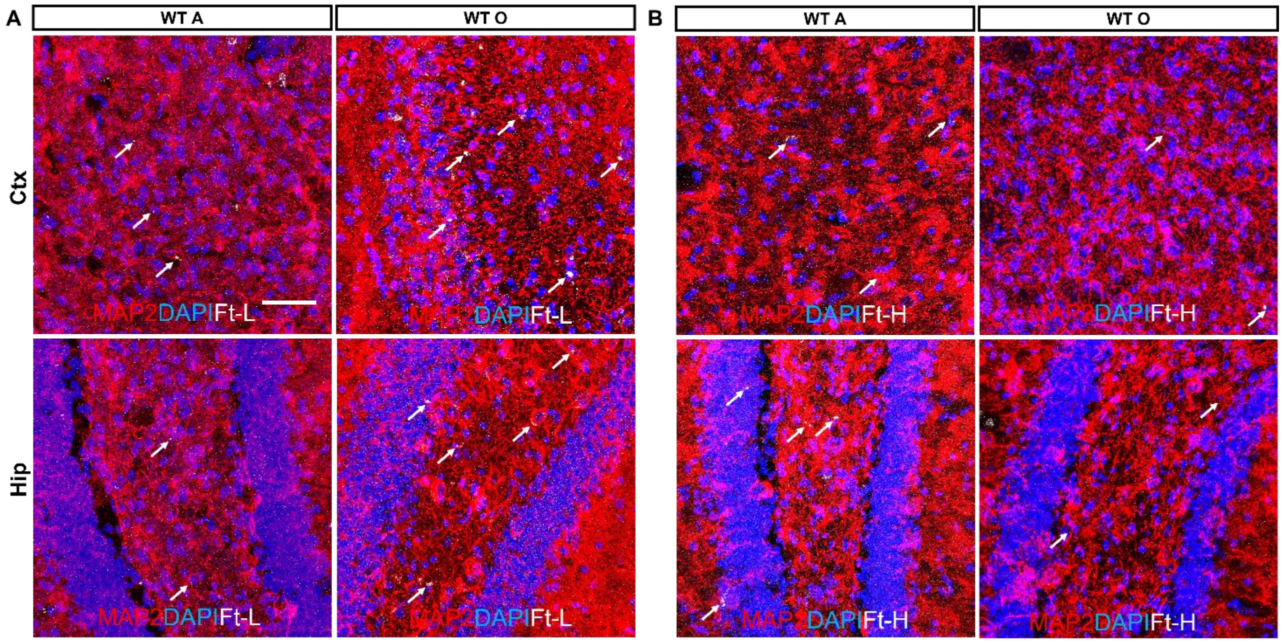

Figure 6S. Ferritin L and Ferritin H proteins cellular allocation in Ctx and Hip.

(**A**) Anti-Ft-L and (**B**) anti-Ft-H in cerebral cortex (Ctx) and hippocampus (Hip) of WT A and WT O mice. Immunofluorescence showing that Ft-L and Ft-H localize both at the level of neuronal soma in the cerebral cortex and hippocampus. To note, also in non-denaturing conditions Ft-H deposits show a reverse trend of expression compared to Ft-L deposits: Ft-H levels are lower in WT A vs WT O; Ft-L levels are higher in WT O vs WT A. Neuronal cells are marked with anti-MAP2 (red), anti-Ft-L or H antibodies (white) are indicated also by arrows and 4,6-diamidino-2-phenylindole (DAPI) (blue) is used to counterstain cell nuclei in cerebral cortex (Ctx) and hippocampus (Hip). Scale bars: 63X.
